# The South to North Variation of Norovirus Epidemics from 2006–07 to 2008–09 in Japan

**DOI:** 10.1371/journal.pone.0071696

**Published:** 2013-08-19

**Authors:** Shinako Inaida, Yugo Shobugawa, Shigeo Matsuno, Reiko Saito, Hiroshi Suzuki

**Affiliations:** 1 Division of International Health (Public Health), Graduate School of Medical and Dental Sciences, Niigata University, Niigata, Japan; 2 Infectious Disease Surveillance Center, National Institute of Infectious Diseases, Tokyo, Japan; 3 School of Nursing, Niigata Seiryo University, Niigata, Japan; Centers for Disease Control and Prevention, United States of America

## Abstract

**Background:**

Norovirus (NoV) is a major cause of gastroenteritis during the autumn and winter seasons in Japan as well as in other temperate climate regions. Most outbreaks are thought to occur by secondary attacks through person-to-person infection by fecal-oral route. Severe cases are found in young children or patients with chronic diseases. Clarifying the patterns of epidemic diffusion is important for considering effective monitoring and surveillance as well as possible prevention.

**Methods:**

We considered the predominant viral genotype from the laboratory result obtained from Infectious Agents Surveillance Report (IASR) of National Institute of Infectious Diseases (NIID). We investigated the increase of NoV cases nationwide for the 2006–07 to 2008–09 seasons using sentinel gastroenteritis data collected from about 3000 pediatric clinics on National Epidemiological Surveillance of Infectious Diseases (NESID) acquired from the kriging method in the geographic information system (GIS).

**Results:**

During these three seasons, the majority of the detected virus was GII.4, which ranged from 60.4 to 88.9%. The number of cases (per sentinel site) at the peak week was 22.81 in the 2006–07 season and it decreased in the following seasons. NoV cases began to increase earlier in the southern areas and gradually extended into the northern areas, similarly, over the seasons. The average period from when the increase of cases was detected in the southern area to when it reached the northern area was 12.7 weeks.

**Conclusion:**

The decrease of the number of sentinel cases at the peak week may suggest the development of herd immunity after a period of high prevalence. Although the NoV epidemic is thought to be associated with cold weather, its cases first increased in the southern area with relatively warm temperature, indicating there are other climate factors involved. Geographic study using the sentinel data could enhance the monitoring and surveillance of and preparedness against epidemics.

## Introduction

Norovirus (NoV) is a major cause of gastroenteritis [1] during the autumn and winter seasons in Japan as well as in other temperate climate regions [2]. NoV patients span all age groups, and the mode of virus transmission is thought to be person-to-person, food-borne, or water-borne [3], [4]. Most outbreaks are thought to occur by secondary attacks through person-to-person infection by the fecal-oral route, contact with contaminated fomites or surfaces, or by inhalation of virus particles from vomitus [5], [6]. The most severe cases are found in young children or patients with chronic diseases and may result in death [7].

NoV is highly communicable with a small infectious dose [8]. Prolonged viral shedding even in asymptomatic people has been reported [7], [9]. NoV shows frequent mutations, and repeated infections could occur because of its molecular changes [10]–[12]. NoV is classified into five genogroups (GI to GV) and multiple genotypes on the basis of the phylogeny of the capsid sequence. In GII, 19 genotypes are recognized, with GII.4 as the leading cause of NoV-associated acute gastroenteritis in humans [13].

In Japan, NoV epidemics are monitored by not only outbreaks but also individual cases through sentinel surveillance, namely, the National Epidemiological Surveillance of Infectious Diseases (NESID) of National Institute of Infectious Diseases (NIID) [14]. The largest epidemic of NoV in the history of national surveillance of gastroenteritis since its launch in 1981 was found in the 2006–07 season and it was caused by the emergence of a GII.4 subtype termed 2006b [15]. Recurring epidemics have led to interest in how the initiation of epidemics occurs and how they are disseminated. Clarifying the patterns of epidemic diffusion is important for considering effective monitoring and surveillance as well as possible prevention. In the present study, we investigated a nation-wide NoV epidemic trend by focusing on the timing of increase of NoV cases for the 2006–07 to 2008–09 seasons using sentinel gastroenteritis surveillance data and the kriging method in GIS (geographic information system).

## Materials and Methods

### 1. Surveillance Data

**Figure 1:**
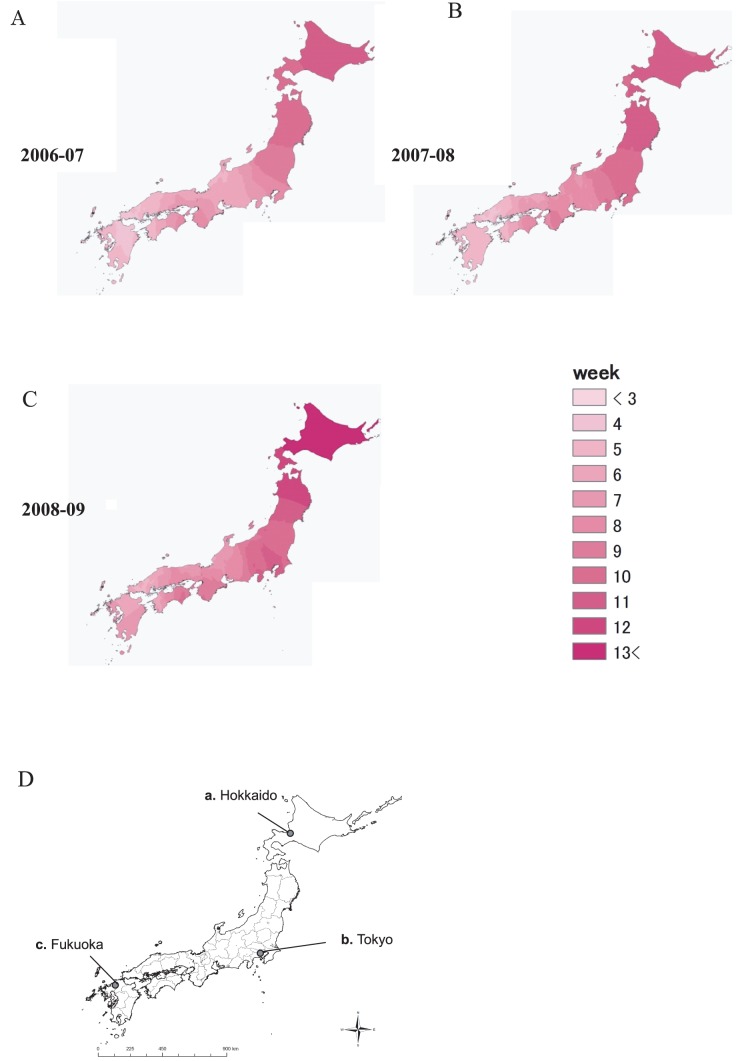
Weekly increase of cases. Spatial distribution of the weekly increase of sentinel cases is depicted by the kriging method (ordinary kriging) at classification colours of 1-week intervals. The first week was defined when more than 4.0 cases per sentinel site was observed in any of the prefectures during the 36^th^ to 8^th^ week period. To obtain coverage of the whole prefecture area in the northern end (Hokkaido and Iwate prefectures) in the kriging method, the locations of the prefecture offices for these two prefectures were modified. (A-C) Map of prefectures, Fukuoka, Tokyo, Hokkaido (D). (Original map data (ESRI Japan)). Sentinel data were obtained from an online web source, “Sentinel-Reporting Diseases (weekly), number of cases and number of cases per sentinel site of the week by prefecture”. (http://www.nih.go.jp/niid/en/survaillance-data-table-english/).

We used sentinel gastroenteritis data obtained from NESID [7], which constitute the weekly number of cases collected from about 3000 pediatric clinics and hospitals covering all of Japan. The data are firstly collected by each physician diagnosing gastroenteritis based on the symptoms of diarrhea, vomiting, and acute abdominal pain (for baby, abdominal pain may not be clear) and reported to the regional health centers. The regional health centers weekly enter the data onto NESID through an online system and the average number of reported cases per sentinel site is used as an index of the epidemic level of the season. The numbers of sentinel clinics are defined by the population size at each regional health center’s administrative sector under 47 prefectures. (Fig. 1D shows the location of prefectures across Japan). The flow of surveillance and reporting system in detail are described elsewhere [14]. The sentinel data also contain age and gender information. The majority of data consist of cases involving patients less than 15 years old because most of the sentinel clinics are pediatric clinics. Although this sentinel surveillance includes other causes of gastroenteritis such as bacterial or other viral causes (i.e. campylobacter, rotavirus), NoV epidemics were also monitored by laboratory confirmation. For laboratory testing, the specimens were routinely collected from all the inpatients and outpatients at hospital admission from about 10% of the sentinel clinics. The results of viral genotyping by RT-PCR were available at most prefectural institutes and reported to the NESID [15], [16]. Some pathogens were also tested by local health centers. All these data include both sporadic cases and outbreaks. We examined the ratio of each viral genotype over 2006 to 2008. We then observed the trend of weekly sentinel case at nation level. The period of data was used from the 36th to the 8th epidemiological weeks, which is approximately from the beginning of September to the end of February and is the estimated NoV epidemic season according to a historical review of the number of detected viruses [17].

### 2. Spatial Trend of the Increase of Sentinel Cases

We investigated the week of increase of cases by using the index of more than 4.0 cases per sentinel site. This case value has been used as a threshold for the observed data over the latest ten years [18]. After the week when the number of sentinel cases recorded was more than 4.0, the NoV sentinel case continued to increase until the peak of epidemic. This threshold has been also used to evaluate the epidemic of the rotavirus, another cause of viral gastroenteritis [19]. The week of the increase in cases was counted for each prefecture when the reported sentinel case met the index during the study period. We applied the kriging method (ordinary kriging), an interpolation method to model the spherical spread by geostatistical estimation of the point based data (prefectural offices) in GIS [20], to the week the cases passed the threshold. The processes were carried out in ArcGIS 9.3 (ESRI) and Spatial Analyst (ESRI) for Windows.

## Results

### 1. Frequency in virus Genotype

The frequency of virus genotypes in the typed result is shown in Table 1 (the number of “not typed” or “genogroup unknown” are not included in the table). Among the typed number of viruses, the percentage of genotypes was 88.9% for GII.4, 2.7% for GII.6, 1.4% for GI.8, and 1.3% for GII.3 in 2006. In 2007 and the 2008, GII.4 again had the highest percentage, but was 78.3% and 60.4%, respectively. In 2007, the next highest were GII.13 at 7.3% and GII.3 at 6.8%, while in 2008 they were GII.3 at 9.3% and GII.6 at 8.4%.

**Table 1:** Distribution of norovirus genotypes in 2006 (A), 2007 (B), and 2008 (C).

	A	(%)	B	(%)	C	(%)
GI.1	3	(0.3)	1	(0.1)	4	(0.6)
GI.2	2	(0.2)	–	–	–	–
GI.3	3	(0.3)	3	(0.4)	2	(0.3)
GI.4	11	(1.1)	36	(4.6)	47	(7.1)
GI.7	1	(0.1)	2	(0.3)	–	–
GI.8	15	(1.4)	6	(0.8)	20	(3.0)
GI.11	2	(0.2)	–	–	–	–
GI.12	–	–	1	(0.1)	–	–
GI.14	2	(0.2)	3	(0.4)	4	(0.6)
GII.1	4	(0.4)	–	–	2	(0.3)
GII.2	10	(1.0)	6	(0.8)	36	(5.4)
GII.3	14	(1.3)	54	(6.8)	62	(9.3)
GII.4	925	(88.9)	619	(78.3)	402	(60.4)
GII.5	–	–	–	–	1	(0.2)
GII.6	28	(2.7)	1	(0.1)	56	(8.4)
GII.7	7	(0.7)	1	(0.1)	–	–
GII.8	3	(0.3)	–	–	–	–
GII.9	5	(0.5)	–	–	1	(0.2)
GII.11	–	–	–	–	–	–
GII.12	–	–	–	–	1	(0.2)
GII.13	6	(0.6)	58	(7.3)	27	(4.1)
GII.14	–	–	–	–	–	–
GII.16	–	–	–	–	1	(0.2)
**total**	1041		791		666	

Data were obtained from an online web source, Infectious Agents Surveillance Report (IASR). http://idsc.nih.go.jp/iasr/virus/graph/rota01-10.pdf.

### 2. National Sentinel Data

**Figure 2:**
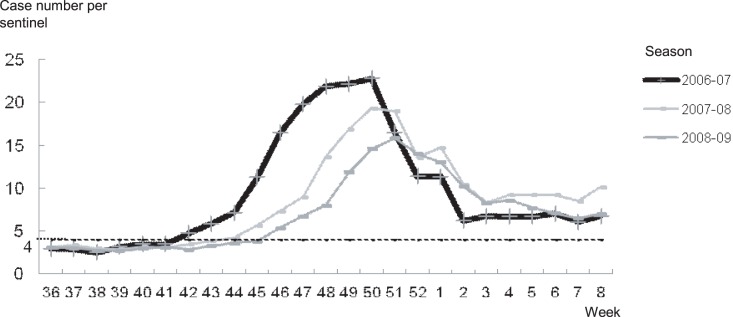
Nationwide weekly number of cases (per sentinel site) of norovirus gastroenteritis from 36th to 8th epidemiological week for the 2006–07 to 2008–09 seasons.

Fig. 2 shows weekly sentinel cases for the three seasons according to the national data. The sentinel cases started increasing over 4.0 cases per sentinel site in the 42^nd^ to 46^th^ weeks in each of the three seasons across the nation. The peaks of epidemic were observed in the 50^th^ week for the 2006–07 to 2007–08 seasons, and in the 51^st^ week for the 2008–09 season. The number of cases at the peak week recorded as 22.81, 19.33, and 15.88 (cases per sentinel site) in the 2006–07 to 2008–09 seasons, respectively.

### 3. Spatial Patterns of the Timing of the Increase of Cases

Spatial patterns of the weekly timing of the increase of cases was investigated by the kriging method in GIS for the three seasons (Fig. 1A-C; the classification colours are set at one week intervals. 1D shows the locations of Fukuoka, Tokyo, and Hokkaido). NoV cases began to increase earlier in the southern areas of Japan and gradually extended into the northern areas. In the 2006–07 season, the increase of cases was first observed in Fukuoka (the southern area) in the 1st week (36th epidemiological week; 2nd week of September), then in Tokyo (the middle area) in the 7^th^ week, and finally in Hokkaido (the northern area) in the 11^th^ week. A similar south to north variation in the increase of cases was also observed in the following two seasons. The average period from when the increase of cases was detected in the southern area to when it reached the northern area was 12.7 weeks, or about 3 months.

## Discussion

In each of the seasons examined, 2006–07 to 2008–09, an increase in NoV cases first occurred in the south and gradually moved north. The average period of this progress was 12.7 weeks, or about 3 months. GII.4 was always the most frequent genotype in each of the three years, but ranged from 60.4 to 88.9%. The reason for this south to north pattern is not certain, although it could suggest climate related effects. The southern area of Japan, such as Fukuoka (except Okinawa), and the middle area, such as Tokyo, lie in the temperate zone and have a yearly average temperature of 17°C. In contrast, the northern area, Hokkaido, lies in the subarctic zone and has a yearly average temperature of 9°C (Fig. S1a shows the monthly averages of daily maximum temperature (2006–2009)). A previous study has concluded that temperature and humidity had highly consistent effects to the NoV epidemic in England and Wales, although a complex interplay may exist between host, viral, and climatic factors [21]. The study has showed that the increases in NoV cases were associated with cold weather, dry temperature, low population immunity, and the emergence of novel genogroup 2 type 4 antigenic variants.

In Japan, the southern, middle, and northern areas have distinguishing climatic factors that could explain the period of the NoV epidemic initiation, which begins in September in the south and reaches the north in December (Fig. S1).

Particularly, when the maximum temperatures were similar in the southern and middle areas between 27.8 to 29.0 degrees and relatively lower in the northern area at under 23.4 degrees (from September onward), differences were found in the relative humidity; it rapidly decreases (−11.3%) in the southern area (Fukuoka) after slightly increasing towards September, and in the middle area (Tokyo) it modestly decreases (−6.7%), and in the northern area (Hokkaido) it slightly decreases (−2.0%) but continues to be high with small increases after October (Fig. S1a, and b). Because NoV cases first increased in the southern area, a warm temperature lowered in association with a relatively rapid change in humidity could be the cause for the increase. The NoV epidemic is also thought to be associated with cold weather [2]. However, most of the epidemic peaks appeared by the end of December, but not in January when the lowest temperature of the year occurs, indicating that there are other climate factors involved [21].

The emergence of a GII.4 subtype, 2006b, brought the largest NoV epidemic. The number of sentinel cases at the peak week was 22.81 (per sentinel site), and it has modestly decreased but remained relatively high in the following two seasons, as 19.33 and 15.88, respectively. 2006b was dominant in the 2006–07 season and remained so in the 2007–08 and the 2008–09 seasons in Japan [15], [22], [23]. The modest decrease of the number of sentinel cases at the peak week may suggest the development of herd immunity after a period of high prevalence, although such immunity maybe limited for short-term [12]. Additionally, 2006b was also detected in the 2005–06 season [22], which would suggest that 2006b remained persistent over the seasons. However, persistency or endemicity of NoV still remains unclear, because NoV cultures are not possible. Meanwhile the south to north pattern of the increase in cases could also suggest a possible migration of the epidemic as well as a dynamic virus transmission beginning from the south area. Although other factors such as difference in population density or standards of medical services may affect the observed geographic pattern of infections, the data obtained in this study do not support this suggestion. Nevertheless timely monitoring should enhance the surveillance of and preparedness against epidemics.

This study has some limitation regarding the data. The sentinel gastroenteritis data are mainly collected from pediatric clinics therefore adult data would have been underreported. Nevertheless, this is the first study to our knowledge that considered the nationwide patterns of NoV epidemics from the national sentinel data.

## Supporting Information

Figure S1 **Monthly averages (2006 -2009) of daily maximum temperature (A) and relative humidity (B) in Hokkaido (North area), Tokyo (Middle area), and Fukuoka (South area).** Data were obtained from Japan Meteorological Agency (http://www.data.jma.go.jp/obd/stats/data/en/smp/index.html).
(DOCX)
