# Supplementary material for: The South to North Variation of Norovirus Epidemics from 2006–07 to 2008–09 in Japan
Source: PLoS One. 2013 Aug 19;8(8):e71696. doi: 10.1371/journal.pone.0071696 (PMC3747177; doi:10.1371/journal.pone.0071696)
Supplement: Figure S1 — Monthly averages (2006 -2009) of daily maximum temperature (A) and relative humidity (B) in Hokkaido (North area), Tokyo (Middle area), and Fukuoka (South area). Data were obtained from Japan Meteorological Agency (http://www.data.jma.go.jp/obd/stats/data/en/smp/index.html). (DOCX) [file pone.0071696.s001.docx]

**Fig. S1. Seasonal climatic variation in Japan**;

Hokkaido (**N**orthern area), Tokyo (**M**iddle area), and Fukuoka (**S**outhern area).

(Average of 3 years, 2006 -2009)

1. Monthly mean daily maximum temperature (^o^C)
2. Monthly mean relative humidity (%)

**a.**

**b.**

**32 33 34 35 36 37 38 39 40 41 42 43 44 45 46 47 48 49 50 51 52 1 2 3 4**

**Month**

**Week**

**South to North initiation of increase of sentinel case**
